# ITO Meta-Absorber-Loaded Conformal UHF Monopole Antenna with Wide-Angel RCS Reduction

**DOI:** 10.3390/ma18061379

**Published:** 2025-03-20

**Authors:** Pan Lu, Jiuhao Gong, Xiaona Liu, Yuanxi Cao, Anxue Zhang, Sen Yan

**Affiliations:** 1 School of Information and Communications Engineering, Xi’an Jiaotong University, Xi’an 710049, China; tianxian769@126.com (P.L.); jiuhao.g@stu.xjtu.edu.cn (J.G.); caoyx@xjtu.edu.cn (Y.C.); anxuezhang@xjtu.edu.cn (A.Z.); 2 Shaanxi Fenghuo Nuoxin Technology Co., Ltd., Baoji 721006, China; lxn18792985715@163.com

**Keywords:** monopole antenna, UHF antenna, meta-absorber, RCS reduction

## Abstract

In this paper, a conformal UHF antenna with a wide-angle radar cross section (RCS) reduction capability is proposed. The radiator of the design is a planar monopole antenna. Since the large physical size of the antenna in UHF band can generate a scatter beam with a large RCS in the high operating frequency of radars and other sensing applications, i.e., the X band, two types of ITO (Indium Tin Oxide) meta-absorber are proposed and loaded onto the monopole antenna to suppress the scatter. For the incident beam around the direction orthogonal to the radiator plane, the periodical meta-absorber can realize around a 20 dB RCS reduction in the X band. The incident wave around the parallel direction of the radiator is absorbed by the taper meta-absorber, which can greatly suppress the surface and then reduce the RCS in the horizontal plane. The combined effect means the antenna can achieve a wide-angle RCS reduction. It should be noted that the antenna can still produce a high-efficiency omnidirectional beam after the lossy meta-absorber is loaded. In our opinion, the advantages of the proposed antenna design, including good radiation performance in UHF band and high RCS reduction in X band, make it a suitable candidate for airborne and drone applications.

## 1. Introduction

UHF antennas play a pivotal role in long-distance radio communication applications, facilitating the transmission of signals across vast expanses with low transmission losses and multipath effects compared with the antennas in microwave and millimeter frequencies [1,2,3]. Several UHF antennas [4,5,6,7,8,9] have been designed with the characteristics of wide operating bandwidth, compact antenna structure, etc. However, due to the fact that the large physical size of the metal radiator for the UHF band can result in a large scattering beam in the high-frequency band, the stealing performance of the antenna is relatively limited, which can greatly impair the stealth performance of the platform.

To overcome this limitation, sub-wavelength periodical structure loaded antennas are proposed with the independent radiation and scattering characteristics manipulating capabilities [10,11,12]. The modified scattering method can be realized by two commonly used technologies, i.e., splitting the scattering beam [13,14,15] and absorbing the incident wave [16,17]. For the first method, a split beam is achieved by fully reflective anti-phase meta-structures, which are loaded around the main radiator of the antenna.

The incident wave will be reflected by a meta-structure with a 180° phase difference and the same magnitude, thus a null will be generated in the incident wave direction for the scattered beam, thus reducing the RCS [18]. The HF/VHF whip antenna in [19] integrates a curved artificial magnetic conductor (AMC) to achieve an RCS reduction in the X band. However, it should be emphasized that this type of RCS reduction only works effectively for monostatic radars, since it cannot cancel the large scattering in the whole space. In contrast, absorber-based RCS reducing antennas can achieve stealing characteristics for both monostatic and biostatic radars [20]. Reference [18] proposes a low-RCS patch array antenna integrated with a resistive loss-based absorbing metasurface. The metasurface exhibits transparency to the antenna’s radiation waves within its co-polarized operating frequency band while demonstrating broadband absorption characteristics for out-of-band or cross-polarized incident waves. However, the fabrication of this metasurface, which incorporates lumped elements, presents certain challenges, and the soldering of lumped resistors increases the manufacturing cost. In contrast, thin-film resistive absorbing metasurfaces utilize materials with electromagnetic loss properties, such as carbon-based materials (e.g., graphite), metal oxides, and lossy dielectrics, to design resonant units. These thin-film designs offer relatively lower fabrication difficulty and cost. In [11], a graphene-based tunable radar absorber with a varied resistance from 120 to 700 Ω/sq is proposed to achieve a wide RCS-reduction bandwidth, but the excessive surface impedance of the absorber makes it unsuitable for conformal integration with UHF antennas, as it will significantly reduce the radiation efficiency of the UHF antenna. In summary, the reported stealth antenna cannot achieve large-angle RCS reduction and high radiation efficiency simultaneously, which are the crucial characteristics of UHF antennas.

Based on the above discussion, an ITO meta-absorber-loaded conformal monopole antenna is proposed to achieve omnidirectional radiation in the UHF band and wide-angle RCS reduction in the X band. The radiator of the antenna is a CPW-fed monopole antenna, which can realize an omnidirectional beam within the operating bandwidth of 0.3 to 0.8 GHz. To suppress the large scattering beam in the X band, two types of meta-absorber are proposed, which can help achieve around a 20 dB RCS reduction in X band.

This paper is organized as follows. Section 2 elaborates on the design process of the stealth antenna, including the design of the monopole antenna, squared ring absorber, and tapered absorber. Section 3 presents the fabrication and experimental evaluation of the antenna, and an error analysis was conducted, demonstrating favorable performance outcomes.

## 2. Antenna Design Procedure

**Figure 1:**
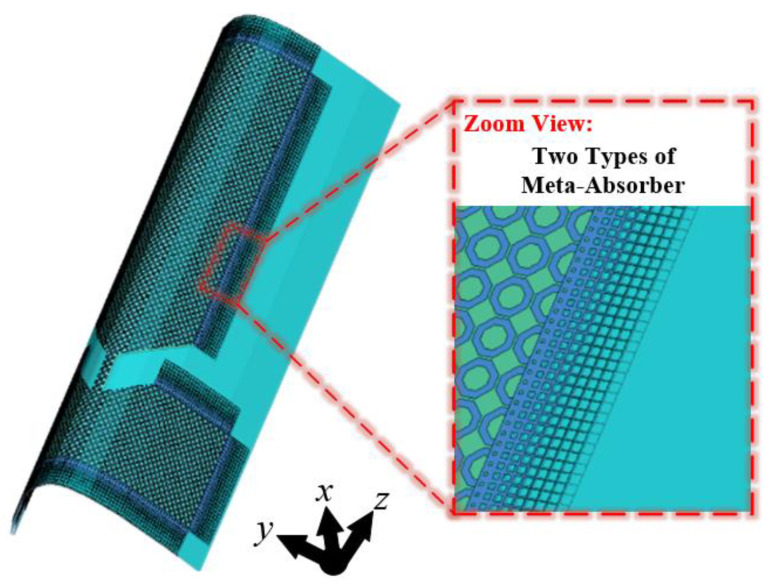
Schematic diagram of the proposed antenna.

The schematic diagram of the proposed antenna is shown in Figure 1. The radiator of the antenna is a CPW fed monopole antenna, which operates in UHF band. Two types of meta-absorber are loaded to suppress the RCS of the antenna in X band. The center squared ring absorbers are used to absorb the normal incident wave, and the side taper absorbers can suppress the surface wave propagation, thus canceling the scatter wave in the reflection at large angles. Due to the ideal flexibility of the ITO board, the antenna can be bent to achieve conformal integration with other platforms. In the simulation setup, the meta-absorbers and the monopole antenna are etched on 0.5 mm glass fiber (dielectric constant: 5.0; loss tangent: 0.0035). The blue dielectric board is a honeycomb structure, which can be regarded as an air gap in simulation. The sheet resistance of the ITO layer is 85 Ω/sq.

### 2.1. The Design and Scattering Characteristics of the UHF Monopole Antenna

**Figure 2:**
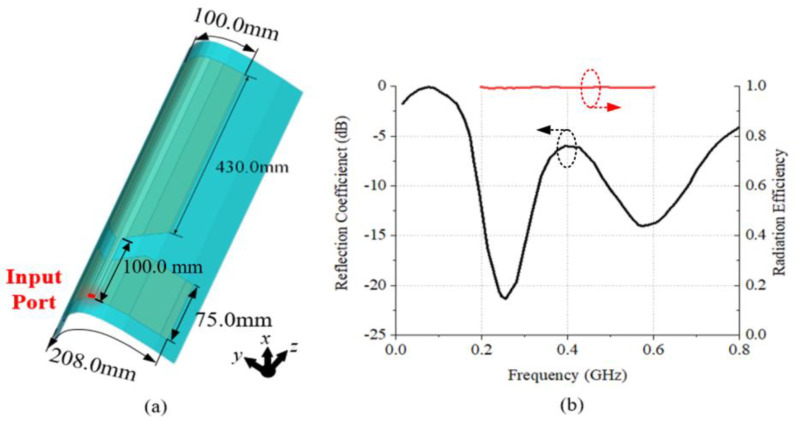
(**a**) The schematic diagram and (**b**) the reflection coefficient (black) and radiation efficiency (red) of the UHF monopole antenna.

The geometry of the external absorber-loaded UHF monopole antenna is shown in Figure 2a, and it can achieve omnidirectional radiation patterns in the xoy plane. The antenna was simulated using CST STUDIO SUITE 2019. The reflection coefficient of the antenna is shown in Figure 2b. It can achieve a −6 dB impedance-matching bandwidth of 0.19 to 0.74 GHz. The radiation efficiency of the antenna is close to 1.0 in this operating band.

**Figure 3:**
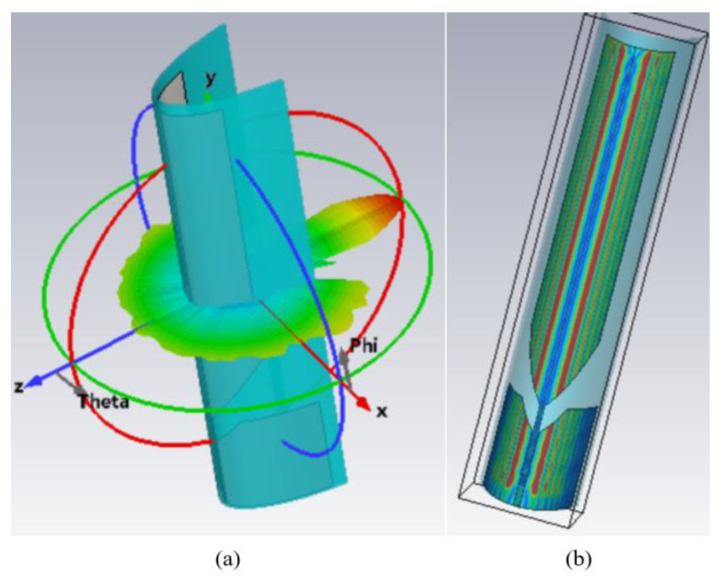
(**a**) The scattering beam and (**b**) the corresponding current distribution of the UHF monopole antenna at 10 GHz.

The scattering beam and its corresponding current distribution for the UHF monopole antenna are shown in Figure 3. The frequency of the incident wave is 10 GHz. It can be seen that the antenna can excite a large scattering value in xoz plane, due to its meter-scale UHF metal radiation element. The current distribution of the antenna shows that the incident wave can generate traveling waves of multiple wavelengths along the radiation element, and also generates large scattering values. Based on these results, it can be concluded that the scattering wave of the antenna is not only generated by the planar reflection, but is also caused by the traveling waves’ propagation, due to its conformal structure. To suppress the RCS of the antenna, the scattering from the reflection wave and the traveling wave should be solved simultaneously.

### 2.2. The Design of the Squared Ring Absorber

**Figure 4:**
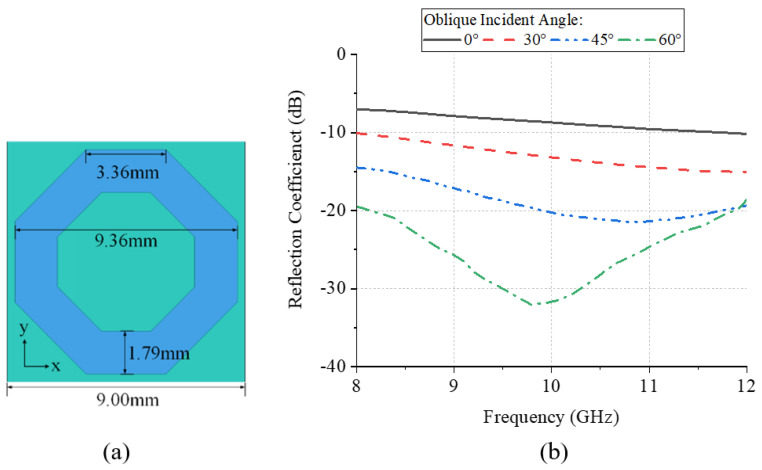
(**a**) The schematic diagram and (**b**) the reflection coefficient with the different oblique incident angles of the squared ring absorber.

For the monopole antenna in Figure 2a, its scattering characteristics can be divided into two parts. The first type of scattering beam is caused by the direct reflected wave, which can greatly increase the RCS. To suppress the large reflection value, a squared ring absorber is proposed, as shown in Figure 4a. The periodical boundary is used to simulate the reflection performance of the absorber unit. It can be seen from Figure 4b that the absorber can achieve a −20 dB reflection coefficient in the X band. For the oblique incident waves, the reflection coefficient is below −10 dB within ±45°.

**Figure 5:**
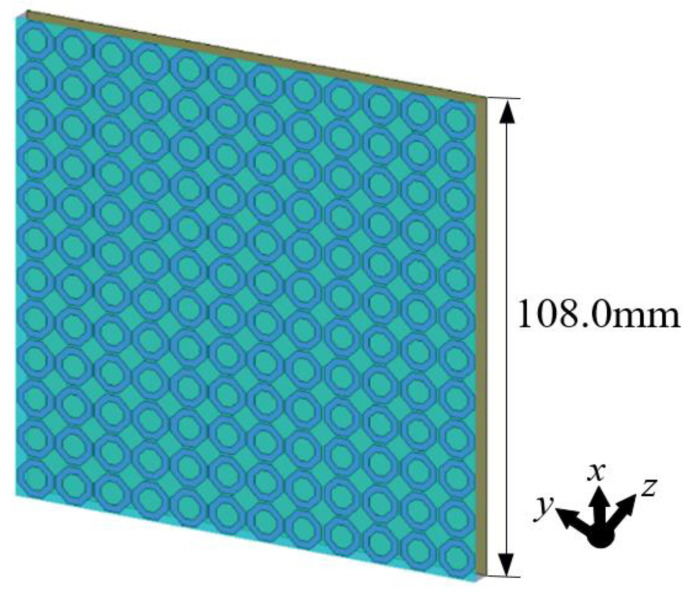
The schematic diagram of the plate with a 12 × 12 unit squared ring absorber array.

**Figure 6:**
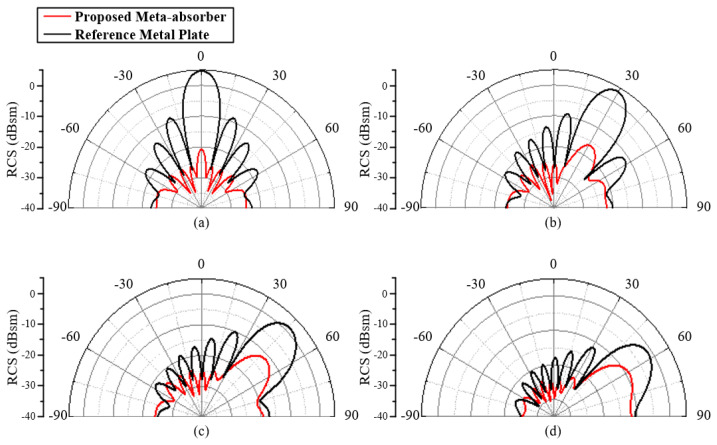
The RCS comparison of the absorber array and the metal plate of the same size. Oblique incident angles of (**a**) 0°, (**b**) 30°, (**c**) 45°, and (**d**) 60°.

To verify the feasibility of the squared ring absorber, a plate array with 12 × 12 units is simulated to give the RCS compared to a metal plate, as shown in Figure 5. The scattering beams with different oblique incident angles are presented in Figure 6. It can be seen from Figure 6a that around a 25 dB RCS reduction is achieved for the normal incident wave, which is a significant value compared with the reference metal plate. For the RCS excited by the oblique incident waves, it also achieves scattering suppression, as shown in Figure 6b–d.

### 2.3. The Design of the Taper Absorber

For the proposed UHF monopole antenna, when the incident wave is normal or nearly normal to the antenna surface, the specular reflection is the dominant source of backscattering. For oblique incidence, the incident wave will produce edge diffraction at the antenna’s edge. Hence, the antenna’s surface will be excited with traveling wave currents. The traveling wave currents propagate to the discontinuous structures or the interfaces of different materials where the surface impedance is discontinuous, resulting in an increased RCS. Therefore, for the normal or quasi-normal incidence with a small incident angle, the squared ring absorber designed in the previous section can effectively suppress the specular reflection and achieve an RCS reduction for the antenna. For the case of oblique incidence, a taper absorber is designed. When it is loaded at the antenna’s edge, it can improve the impedance discontinuity between the antenna’s edge and the medium and, to some extent, suppress traveling wave scattering and absorb edge-diffracted waves. Thus, it can achieve the RCS reduction for the antenna at large oblique incidence angles.

**Figure 7:**
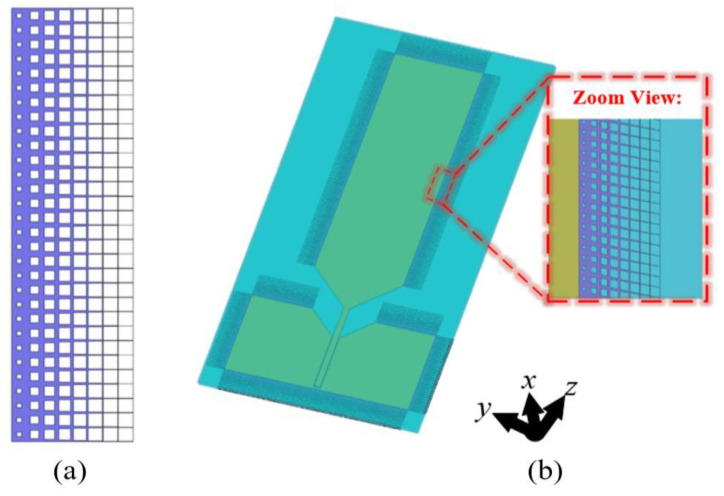
Schematic diagram of (**a**) taper absorber and (**b**) loading position of taper absorber.

As shown in Figure 7, the taper absorber is composed of eight gradient units, and the same units are arranged repeatedly in the other direction. The material of the entire structure is still ITO film. Each unit is etched with a square hole with a gradually increasing side length. The side length of each unit is 2.5 mm, and the side lengths of the holes are 0.75 mm, 1.40 mm, 1.70 mm, 1.95 mm, 2.15 mm, 2.30 mm, 2.40 mm, and 2.45 mm, respectively. This is to achieve a gradual surface impedance transition from the antenna edge to the medium.

**Figure 8:**
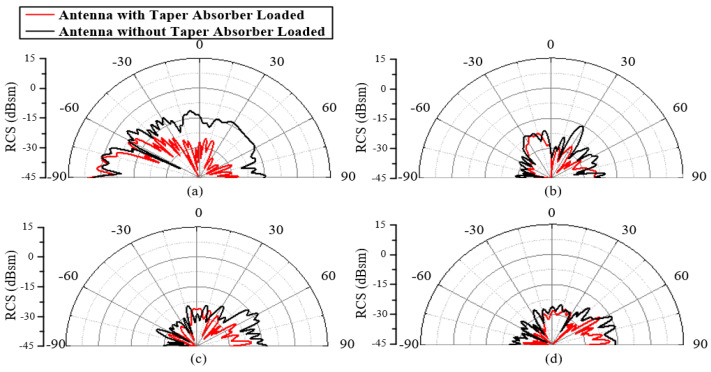
An RCS comparison of a monopole antenna with/without a taper absorber loaded. Oblique incident angles of (**a**) 90°, (**b**) 60°, (**c**) 30°, and (**d**) 0°.

Next, to verify the RCS reduction effect of the taper absorber-loaded monopole antenna, 10 GHz plane waves with incident angles of 0°, 30°, 60°, and 90° were used to simulate the scattering performance. The scattering patterns of the stealth antenna and the original antenna are shown in Figure 8. It can be observed that the stealth antenna achieves a certain reduction in RCS in the backscatter direction for the normal incident wave. When the incident angle increases from 30° to 90°, the incident elevation and azimuth angles of the plane wave relative to the antenna surface are both non-zero. Under these conditions, the stealth antenna still exhibits a certain RCS reduction effect in the backscatter direction. Under the combined effect of the two types of absorbers, the proposed conformal antenna can achieve a good RCS reduction effect within the different radar detection directions.

## 3. Fabrication and Measurement

**Figure 9:**
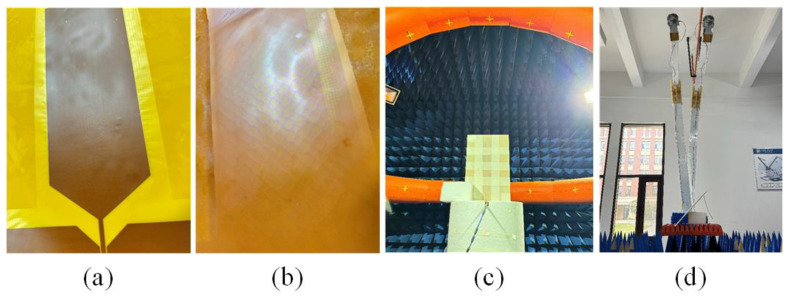
Schematic diagram of the fabricated (**a**) monopole antenna and (**b**) ITO meta-absorber; measurement environments of (**c**) radiation and (**d**) scattering performances.

Based on the design procedure in Section 2, an antenna prototype was fabricated and measured. The layers of the monopole antenna and the ITO meta-absorber are shown in Figure 9a,b. The S-parameters of the proposed monopulse antenna array were measured utilizing an AV3672E Keysight Network Analyzer. The radiation performance of the antenna was tested in a multi-probe anechoic chamber, as illustrated in Figure 9c. The scattering characteristics of the antenna were measured using a material reflectivity and transmissivity testing system. The testing environment for the scattering characteristics is shown in Figure 9d. The antenna under test was placed horizontally on the test platform, with the transmitting and receiving horn antennas positioned at the 0° azimuth of the testing system. The difference in reflectivity between the stealth antenna and the original antenna was measured, and this value represents the radar cross section (RCS) reduction in the stealth antenna relative to the original antenna.

It should be noted that both the antenna and the ITO-resistive film are printed on a polyimide substrate sheet, which is around 150 g. The absorber is very lightweight, and the added weight is minimal compared to the original weight of the aircraft’s antenna, having almost no impact on the overall weight of the airframe.

**Figure 10:**
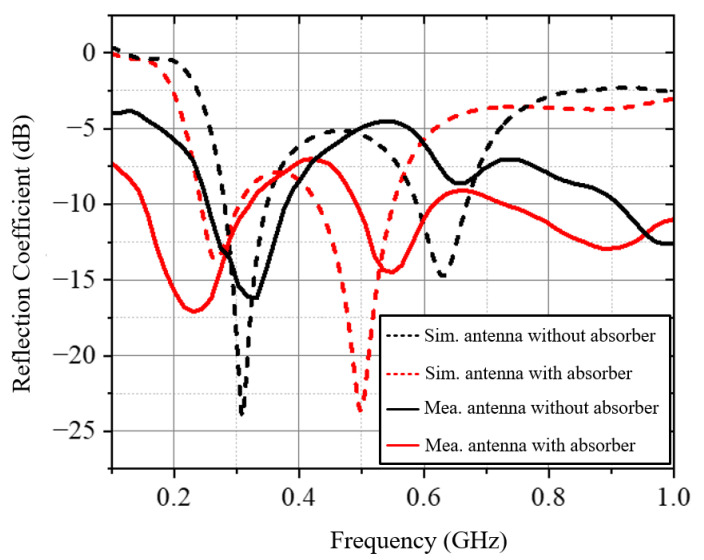
The simulated and measured S-parameters of the antenna with/without a meta-absorber loaded.

**Figure 11:**
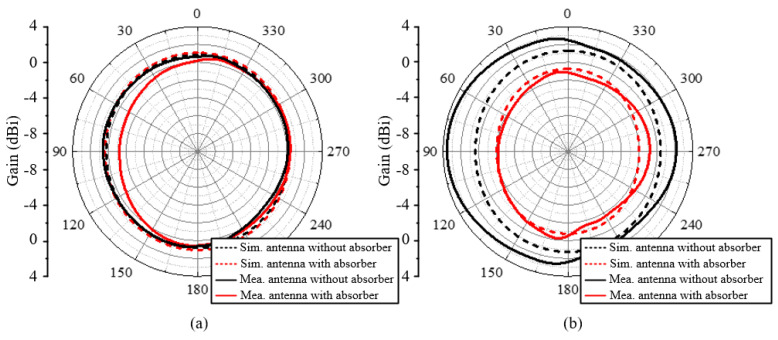
The simulated and measured (**a**) 0.45 GHz and (**b**) 0.55 GHz radiation patterns of the antenna with/without a meta-absorber loaded.

The simulated and measured S-parameters of the antenna with/without a meta-absorber loaded are compared in Figure 10. It can be seen that the results show reasonable agreement in the operating bandwidth. After loading the meta-absorber, the thin-film resistors placed around the antenna increase the overall equivalent size of the antenna, causing the resonant frequency to shift further toward the lower frequency range. Additionally, the in-band matching performance was improved. As shown in Figure 10, the measured resonant frequency of the antenna exhibits a slight deviation from the simulation results, and the depth of resonance also differs to some extent. The source of this error may be attributed to discrepancies between the dielectric constant of the honeycomb substrate used in practice and the values set in the simulation. Furthermore, during the soldering of the coaxial feed line, a window was cut into the fiberglass substrate at the bottom layer of the antenna to expose the coplanar waveguide’s signal line and ground plane for soldering. This process may also have contributed to the differences between the measured and simulated results. Nevertheless, the overall error is within an acceptable range, and the antenna’s matching performance still meets the operational requirements. The overall results sufficiently demonstrate the feasibility of the proposed design. The radiation patterns of the antenna are presented in Figure 11. The frequencies of the beams are 0.45 and 0.55 GHz. It can be observed that the antenna can achieve a good omnidirectional radiation pattern with an out-of-roundness below 4 dB. The maximum gain variation caused by the meta-absorbers is 4.2 dB, proving that meta-absorbers have a negligible impact on the radiation performance in UHF band.

**Figure 12:**
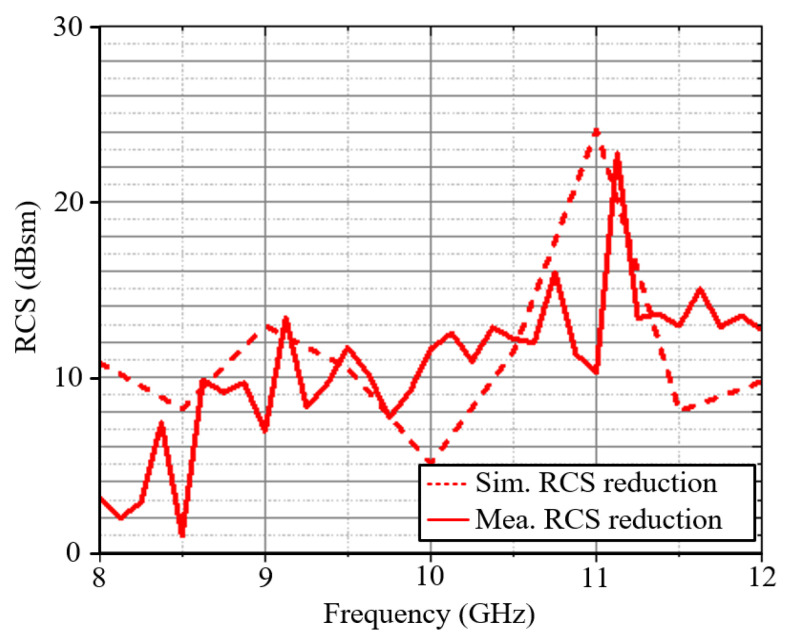
The simulated and measured RCS reductions for the proposed antenna.

The simulated and measured RCS reduction is shown in Figure 12. In the X-band, the antenna achieves an RCS reduction of approximately 10 dB. Although there is some degradation for the measured results around 8 GHz, we believe that the overall results are sufficient to demonstrate the feasibility of the design. Table 1 compares the effects of RCS realized by similar metasurface structures in recent articles. Based on the comparison, this antenna has advantages such as a relatively small size, a wide-angle RCS reduction, and an excellent RCS performance.

## 4. Conclusions

A conformal UHF antenna with a wide-angle RCS reduction capability was successfully developed. The antenna employs a planar monopole radiator and integrates two types of ITO meta-absorbers to achieve RCS reductions in high-frequency applications. The design effectively reduces RCS across multiple angles while maintaining high-efficiency omnidirectional radiation performance. The proposed antenna is suitable for airborne and drone applications due to its combined advantages of good radiation performance in the UHF band and significant RCS reduction in the X band.

## Figures and Tables

**Table 1:** RCS comparison of different metasurfaces.

Ref.	Flexibility	Polarization	Operating Bandwidth	Oblique Incidence Effective	Antenna Unit Size (*λ*_0_)	RCS Reduction
[21] 2023	×	LP	14.2–15.5 (87.5%)	×	2.48 × 2.48 × 0.09	9.31–28.22 (>6 db)
[22] 2023	×	LP	4.8–6.0 (22%)	√ (Narrow)	1.15 × 1.15 × 0.13	4.3–13.5 (>7 db)
[23] 2022	×	LP	9.5–10.15 (6.6%)	√ (Narrow)	2 × 2 × 0.8	6–18 (>5 db)
This work	√	LP	0.19–0.74 (85.2%)	√ (Wide)	0.822 × 0.434 × 0.09	8–12 (>10 db)

*λ*_0_ is the free-space wavelength at the center of the operating frequency.

## Data Availability

The original contributions presented in the study are included in the article, further inquiries can be directed to the corresponding author.
